# Milk Granules

**Published:** 1875-04

**Authors:** 


### MILK GRANULES.

This term describes more clearly than the name given by the proprietors, the character, composition, and appearance of a preparation for infants, which Messrs. Hall & Ruckle have from time to time advertised in our pages, under the title of "The Van Deren Remedy." Before these little round grains of concentrated food were given to the public, we were fully informed concerning their composition, and our judgment was solicited as to the value of the article. We felt satisfied that it would prove very useful in all cases of bowel-complaint, cholera infantum, and deficient nutrition in young children. Acting upon this belief, we recommended its use in a large number of bad cases, and the effect was almost magical. We are of the opinion that all our readers who are raising young children, would do a wise thing to procure a fifty cent bottle of these granules and employ them in all severe cases of bowel disorder. We have little faith in patent nostrums and cure-alls, but our experience with this article has been large, and we know we are doing our friends a genuine service in recommending it. It is, in fact, a food refined to the highest degree of concentration; and, while assimilating readily, it has the singular power of inducing the assimilation of such other nourishment as may be taken. Its preparation, as we have witnessed it, is one of the most skillful acts of modern chemistry. Its general use would save thousands and tens of thousands of lives of little ones this year. We say this freely and cheerfully, with no desire to benefit Messrs. Hall & Ruckle, who are very worthy gentlemen, but with an earnest hope that some little one may be saved from death through the words which we utter so cordially and earnestly.

---

ALCOHOL.—Alcohol has lately been recommended, particularly with children, as the most rapid and effective means of alleviating the pain of burns. The affected part is either simply covered loosely with an alcoholic compress, or is bathed with alcohol, when the pain instantly disappears, but returns when the application ceases. It must, therefore, be continued for one to two hours, and then be repeated at longer intervals, until the reddened epidermis is bleached and shriveled, or until any blisters that may have formed have opened and discharged, which will take place in from six to twelve hours. Care must be taken, especially where the surface to be treated is large, that the vapor of the alcohol does not affect the patient, as it is very easy to get tipsy on the alcoholic fumes. The alcoholic application undoubtedly relieves the pain speedily, but scarcely quicker than the use of very cold water. We have found sulphuric ether to answer a better purpose than alcohol, as the evaporation is more rapid, securing a speedier diminution of the temperature. Doubtless the rapidly evaporating fluids distilled from petroleum, and known as benzole or naphtha, would prove quite as valuable as any other application.

---

In these hard times, he who can sit on a stone and feed himself had better not move.

